# P306: Knowledge and compliance to standard precautions among brazilian manicures and pedicures

**DOI:** 10.1186/2047-2994-2-S1-P306

**Published:** 2013-06-20

**Authors:** JL Garbaccio, AC Oliveira, AO de Paula

**Affiliations:** 1 Nursing, Federal University of Minas Gerais, Belo Horizonte, Brazil

## Introduction

The nature of the daily tasks of beauty establishments facilitates the transmission of microorganisms, especially in Brazil, where people remove cuticles from the nails. Therefore, standard precaution behavior, including self protection equipment, is important to minimize the contact with biological materials.

## Objectives

This study assessed the knowledge and the compliance of manicure/pedicure to the standard precaution recommendations.

## Methods

A Survey was conducted with a random sample of 200 professionals, older than 18, in 200 beauty salons in Belo Horizonte, Brazil. A questionnaire was applied (Jul/12-Jan/13) to obtain demographic information and the standard precaution recommendations of these professionals. The results were analyzed by the software SPSS. The study was approved by the Ethics Committee of the Federal University of Minas Gerais.

## Results

All the professionals interviewed were women, had an average age of 30, more than 10 years of experience (11%), work more than 8 hours per day (57%), being less than a year in that salon (34%). As for education 55% had completed high school, 54% had taken some courses in the field, yet 38% became manicure/pedicure by her own initiative. Moreover, 76% had never received any training in biosafety. With respect to the compliance and knowledge questions, 32% considered the use of self protection equipment (SPE) important and they claim they do so (p=0,24). However, most manicures/pedicures (67%) said they did not use any SPE despite defending the use of gloves (93%), mask (59%) and goggles (27%), nonetheless 69% revealed that have had injuries during service. The highest adherence to SPE was for gloves (31%) followed by mask (16%). From those who use SPE, 22% said they do that for all clients and 8% only when she notices that the client has some disease. Even though for the question regarding knowledge, 82% declared that gloves should be used in all procedures. The main reasons for non-use of SPE were the discomfort of using them (63%) and the perception of no risk of contact with blood and injuries (11%).

## Conclusion

Due to the risk of microbial dissemination, educational actions should be implemented to broaden and deepen the knowledge of professionals regarding the recommendations for biosafety, especially for the use of SPE.

## Funding Agent

Fapemig nº 00340-11

## Disclosure of interest

None declared

